# Association between terbinafine hydrochloride and sperm DNA fragmentation - case report

**DOI:** 10.5935/1518-0557.20210093

**Published:** 2022

**Authors:** Andressa Moreira Giusti, Gabriela Reif, Danilo Modafaris Araújo, Alfred Paul Senn, Vera Lucia Lângaro Amaral

**Affiliations:** 1 Universidade do Vale do Itajaí, Itajaí - SC, 88302-901, Brazil; 2 University of Geneva, Department of Genetic Medicine & Development, Geneva, Switzerland

**Keywords:** sperm analysis, human, terbinafine, antifungal, case report, DNA fragmentation

## Abstract

**Objective:**

To present the case of a man with normozoospermia and a high level of fragmented spermatozoa, which origin seems to be associated with long-term treatment with terbinafine hydrochloride.

**Case description:**

A 20-year-old male healthy patient, with no history of disease and addictions, used an antifungal (terbinafine hydrochloride) for one year to treat a toenail. During this treatment, he participated in a study to evaluate a method of sperm DNA fragmentation analysis. He had 99% fragmented sperm, primarily attributed to prolonged abstinence. The samples that were analyzed later indicated that the high fragmentation could be associated with the antifungal treatment and that with a 2-day abstinence and absence of treatment the fragmentation rate was again comparable with that of fertile men (15%).

**Conclusion:**

Terbinafine hydrochloride is likely to cause problems in male fertility, mainly affecting DNA sperm integrity. Further studies are needed to confirm this observation and to determine at what level of the genitourinary tract the alteration of DNA occurs.

## INTRODUCTION

Terbinafine hydrochloride is a drug indicated in the treatment of several fungal infections of the skin and nails (AbdelSamie *et al*., 2016; Kanakapura & Penmatsa, 2016; Kuminek *et al*., 2013). This drug inhibits the squalene-epoxidase enzyme, responsible for the biosynthesis of ergosterol, an essential sterol of the plasma membrane of fungi with functions like those of cholesterol in animal cells. The decrease of ergosterol modifies the membrane permeability, causing lysis and cell death (Petranyi *et al*., 1984; Stütz & Petranyi, 1984).

Terbinafine hydrochloride is part of the list of essential drugs of the World Health Organization (WHO, 2019) and its package insert indicates possible side effects, but it does not provide relevant information on fertility in animals or humans (CRISTALIA - Produtos Químicos Farmacêuticos Ltda., 2017). However, some studies suggest that certain antifungals can impact spermatogenesis (Millsop *et al*., 2013; Pasqualotto, 2007; Semet *et al*., 2017; Stedile, 2014).

To evaluate male fertility, a set of tests are performed, among which sperm analysis is the most important, and it includes evaluations of spermatozoa concentration, motility, vitality, and morphology (WHO, 2010). The correlations between the values of these parameters and the occurrence of pregnancy are complex and the diagnosis of the presence of a "male factor" in infertility requires the evaluation of a specialist (Cooper *et al*., 2010; Guzick *et al*., 2001; Pasqualotto, 2007; Pasqualotto *et al*., 2005).

Among the factors involved in successful fertilization and embryonic development, sperm DNA fragmentation is associated with increased miscarriage (Gosálvez *et al*., 2013; McQueen *et al*., 2019; Zhang *et al*., 2015), but there is still no clinically recognized threshold for choosing the best medically assisted procreation treatment (Vandekerckhove *et al*., 2016; Zhang *et al*., 2015). The causes of DNA fragmentation are associated with several factors such as protamine/chromatin deficiency, failure to repair breaks in DNA strips, and exposure to reactive oxygen species (ROS) during sperm transport through the genitourinary tract (Chohan *et al*., 2006; Evenson, 2016; Gosálvez *et al*., 2011; Practice Committee of the American Society for Reproductive Medicine, 2013; Rex *et al*., 2017; Sakkas & Alvarez, 2010; Zini & Libman, 2006). Thus, in recent years, spermatic DNA fragmentation testing has become an important biomarker for male infertility (Ribas-Maynou *et al*., 2013).

The objective of this report is to present a case of a man with normozoospermia and a high level of fragmented spermatozoa, whose origin appears to be associated with antifungal treatment.

## CASE DESCRIPTION

This is a male, 20-year-old healthy patient, with no history of relevant diseases and no addictions. He reported suffering an injury during a sports practice at the end of 2016, followed by the nail loss of the left foot big toe. After anamnesis and evaluation of the lesion, performed by a dermatologist in 2018, the patient started oral treatment with terbinafine hydrochloride (500 mg/day, for 1 year). During the treatment, the patient did not have complications or side effects caused by the medication and did not use any other medication.

In May 2019, the patient initiated his participation, voluntarily, in an academic study of sperm DNA fragmentation analysis, approved by the Research Ethics Committee. Two seminal samples were analyzed on May 9, 2019, with an interval of one hour between collections. Both samples were classified as teratozoospermic (WHO, 2010) and had sperm DNA fragmentation indexes (DFI) of 99% and 100%, respectively. On May 20, 2019, eleven days after the first evaluation, a new analysis was performed and the DFI was 84%.

To determine if the high fragmentation rate could be due to the antifungal treatment, the patient decided to voluntarily suspend it, since the toe treatment had been successful. A new semen analysis was performed on September 10, 2019 and showed a DFI of 15% without major changes of the sperm parameters. On December 4, 2019, after six months and fourteen days without the use of terbinafine hydrochloride, a new analysis was performed. He was then classified as normozoospermic and presented a DFI of 44%. It is worth mentioning that, in the week before the examination, the patient used an antibiotic (Cephalexin 500 mg, 4 pills/24h), to treat a skin infection.

**Figure 1:**
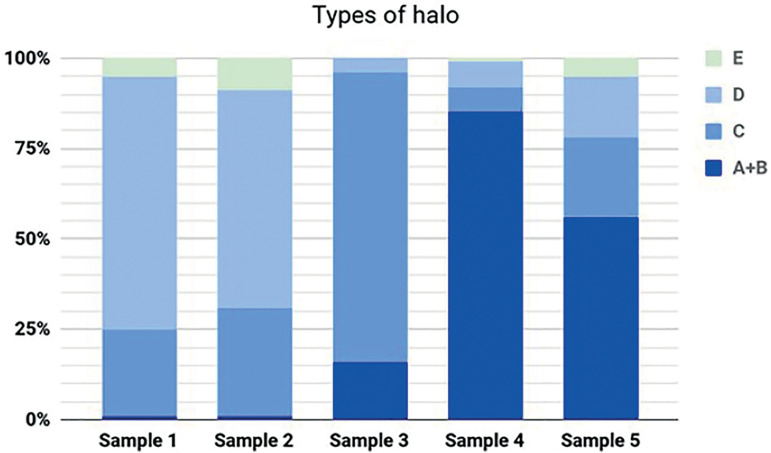
Percentage of the types of halos observed in the samples submitted to the DNA fragmentation test. Types (A+B): medium to large halo, (C): small halo, (D): no halo, (E): degenerated nucleus.

All the data obtained in the sperm tests are presented on Table 1, the halo types (%) are shown in Figure 1.

**Table 1.** Data obtained after seminal analysis and spermatic DNA fragmentation test. Samples with an asterisk (*) were collected during the treatment period with Terbinafine Hydrochloride. The leukocytes were differentiated by the peroxidase technique (ENDTZ) (Shekarriz et al., 1995).

Analysis	Samples				
	1*	2*	3*	4	5
Date of analysis	09/05/2019	09/05/2019	20/05/2019	10/09/2019	04/12/2019
Abstinence (days)	30	1 hour	2	2	2
Volume (mL)	5.2	3.4	2.5	2.5	2.7
Sperm Concentration (10^6^/mL)	207	70	95.5	55	35.7
Round cell concentration (10^6^/mL)	12.9	1.5	5	1.8	2.6
Leukocyte concentration 10^6^/mL)	2.4	0.9	0	0.04	0
Progressive and (total) motility (%)	53 (67)	30 (44)	51 (78)	45 (70)	61 (79)
Vitality (%)	70	83	93	95	80
Normal morphology (%)	3	3	3	4	4
DFI (%)	99	99	84	15	44

## METHODS

Terbinafine hydrochloride (C21H26ClN) was administered orally (2 tablets of 250mg/24h, Funtyl^®^, Cristália, Itapira-SP, Brazil). The protocol used to detect DNA fragmentation was developed and validated during the execution of a research project and was based on the evaluation of spermatic DNA dispersion after the denaturation of nuclear proteins (Fernández *et al*., 2003). Briefly, an aliquot of semen (60 µL), previously diluted to a concentration of sperm near 20 x 10^6^/mL was mixed with 140 µL of a 1% agarose solution (Sigma Aldrich), maintained liquid at a temperature of 90°C. From this mixture, 30 µL was deposited on a microscope slide, pre-treated with a 0.65% agarose. The sperm mixture was immediately covered with a coverslip (22x22 mm) and the slide was cooled to 4°C for five minutes before careful removal of the coverslip. The area containing the agarose was then treated sequentially with a denaturing solution (0.08N HCl) for 7 min, a lysis solution (Tris-HCl 0.4M, Dithiothreitol 0.8N, SDS 1%, EDTA 50mM, pH 7.5) for 10 min, a solution of lysis 2 (Tris-HCl 0.4M, SDS 1%, NaCl 2M, pH 7.5) for 5 min, a phosphate buffer (pH 7.3, Ingámed, Brazil) for 5 min, alcohol 70% and 100% for 2 min, before staining with the Panotic kit (Laborclin, Pinhais-PR, Brazil) without prior fixed treatment.

**Figure 2:**
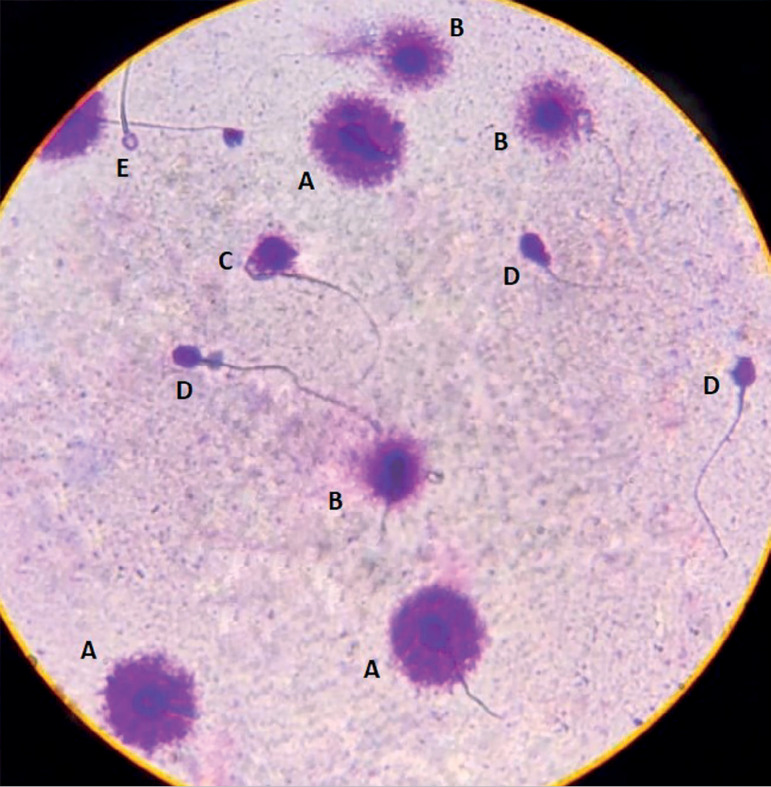
Sperm of the fourth seminal sample with types of halo (A) - nucleus with large halo of dispersion of DNA; (B) - medium size; (C) - small size; (D) - nucleus without halo; (E) - degenerated. (DFI=15%).

The slides were studied under a microscope (400x), under bright field illumination. The spermatozoa (N=200) were classified according to the size of the halos formed around the head (Fernández *et al*., 2005), as (A) - nucleus with a large halo of DNA dispersion; (B) - nucleus with a medium-sized halo; (C) - nucleus with a small-sized halo; (D) - nucleus without halo; (E) - nucleus without halo and degenerated (Fig 2). The DNA fragmentation index (DFI) was calculated as:


$$\mathrm{DFI}\left(\%\right)=\left(C+D+E\right)/\left(A+B+C+D+E\right)*100$$

Each batch of lysis solutions 1 and 2 produced was validated with a positive control, using semen treated with an iso-osmotic solution containing hydrogen peroxide (0.5%) to induce DNA fragmentation through the release of ROS and confirm the absence of halo formation (Cicaré *et al*., 2016).

## DISCUSSION

The case presented hereby describes a temporal association between long-term oral treatment with terbinafine hydrochloride and the fragmentation of spermatic DNA of a healthy young man. This occurrence, according to our knowledge, has not yet been described in the literature. The interruption of treatment induced a reduction of fragmentation to values normally seen in fertile men (Table 1).

The effects of terbinafine and ketoconazole on the testicular-hypophysis axis in young men were seen after short-term treatment (Nashan *et al*., 1989). They reported that while the use of ketoconazole showed a decrease in serum testosterone levels and an increase in 17-hydroxyprogesterone levels, the same was not reported with terbinafine, which did not cause any effect on testosterone, as well as on hydroxyprogesterone and LH levels. This may explain the absence of alterations in the sperm parameters, since terbinafine does not seem to interfere directly in spermatogenesis, or indirectly through endocrine hormones. In our case, the long-term treatment with terbinafine showed no effects on spermatogenesis and motility.

In sample 1, we found (Table 1) a high concentration of round cells (12.9 x 10^6^/mL), associated with a high concentration of leukocytes (2.4 x 10^6^/mL) and a high rate of sperm with fragmented DNA (DFI=99%). The patient also reported a long abstinence (30 days), which led the laboratory to request a second sample an hour later (Sample 2). The fragmentation rate remained very high (DFI=99%), but the leukocyte count decreased to 0.9 x 10^6^/mL. The patient was then instructed to maintain an ejaculatory frequency of 2 days and return for a third analysis about 10 days later. In this third sample, the leukocyte concentration decreased to 0 and the total mobility increased to 78%, but the DNA fragmentation remained high (84%). Only after the prolonged absence of terbinafine hydrochloride administration did the fragmentation rate decrease to 15% (Sample 4). A final semen collection was performed three months later, almost six months after the antifungal treatment was discontinued (Sample 5). The concentration, motility, and sperm morphology were still within normal limits, but the fragmentation rate had increased again (DFI=44%). It is worth noting that the patient was treating a skin infection in the week before producing Sample 5. Infections are known to cause damage to the sperm DNA, due to increased circulation of ROS, produced by the numerous leukocytes, in addition to the rise in temperature that can occur at times of fever.

While treatment with terbinafine hydrochloride seems to be associated with very high values of sperm DNA fragmentation, the increase in fragmentation rates after 6 months without treatment seems to indicate that terbinafine is not the only factor involved. We know that ROS are produced under several conditions (Wright *et al*., 2014), such as advanced age (Nguyen-Powanda & Robaire, 2020), cancer (Loft & Poulsen, 1996), varicocele (Abdelbaki *et al*., 2017), infections, and inflammations in the urogenital tract (Hamdi *et al*., 2020; Potts & Pasqualotto, 2003), fever and hyperthermia (Rao *et al*., 2015), duration of abstinence (Comar *et al*., 2017), smoking (Loft & Poulsen, 1996) and exposure to pesticides (Khan *et al*., 2015; Zamkowska *et al*., 2018). In our case, it is not clear which of these factors may, besides terbinafine, also have contributed to DNA fragmentation - prolonged abstinence being one of them.

When the abstinence was prolonged, the effects of DNA fragmentation were more visible. Decreasing the abstinence, younger spermatozoids are ejaculated, and are less affected by fragmentation. This is indirect proof that spermatogenesis and testicles are not affected by terbinafine, and the effect of terbinafine may be caused at the level of the epididymis (Okada *et al*., 2020). Therefore, it is important to investigate further the likely mode of action of terbinafine on spermatozoa and DNA fragmentation. The first hypothesis would be that it exerts a direct effect, penetrating the spermatozoa and affecting the DNA; the second hypothesis is about the indirect effect, in which terbinafine causes an effect on other cells that will cause the fragmentation, releasing ROS, for example.

The case reported hereby should serve as a warning to healthcare professionals, both when prescribing long-term terbinafine treatment and when initiating medically assisted procedures, especially since no sperm parameters seem to be affected by this molecule. Likely, the direct or indirect effect of terbinafine on sperm DNA fragmentation will only be visible during pregnancy, resulting in spontaneous abortion

## CONCLUSION

We report an association between terbinafine hydrochloride treatment and an increase in sperm DNA fragmentation, while sperm parameters were not significantly affected. This effect increases with prolonged abstinence, which suggests an action in the transit of the epididymis. The use of this drug could cause problems in male fertility, the biomolecular mechanisms being unknown for the time being, which suggests that more studies need to be conducted in this field.
